# Supplementary figures and images for: Normal Values for the fT3/fT4 Ratio: Centile Charts (0–29 Years) and Their Application for the Differential Diagnosis of Children with Developmental Delay
Source: Int J Mol Sci. 2024 Aug 6;25(16):8585. doi: 10.3390/ijms25168585 (PMC11354987; doi:10.3390/ijms25168585)

**fT3/fT4 ratios in normal females at 0–29 years**

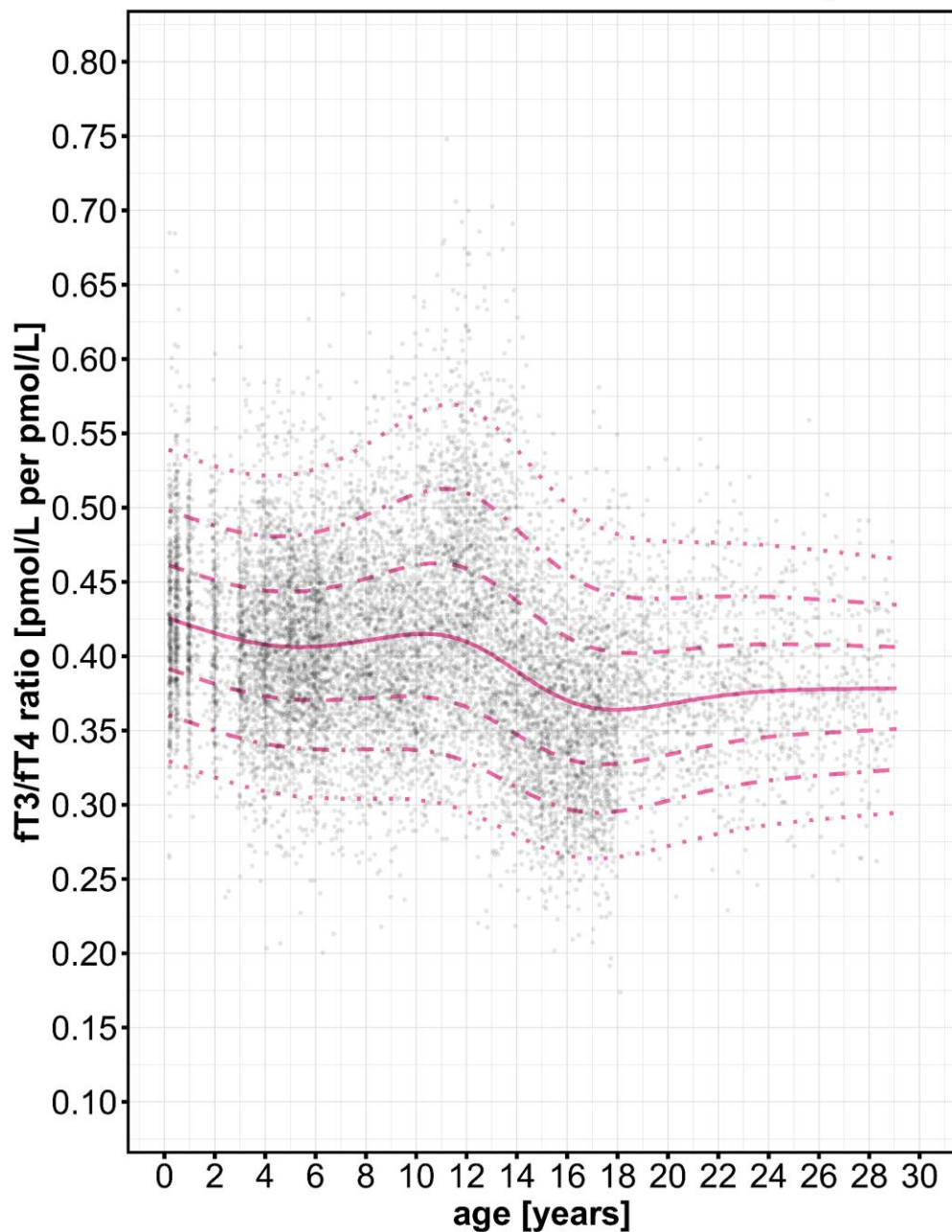

**fT3/fT4 ratios in normal males at 0–29 years**

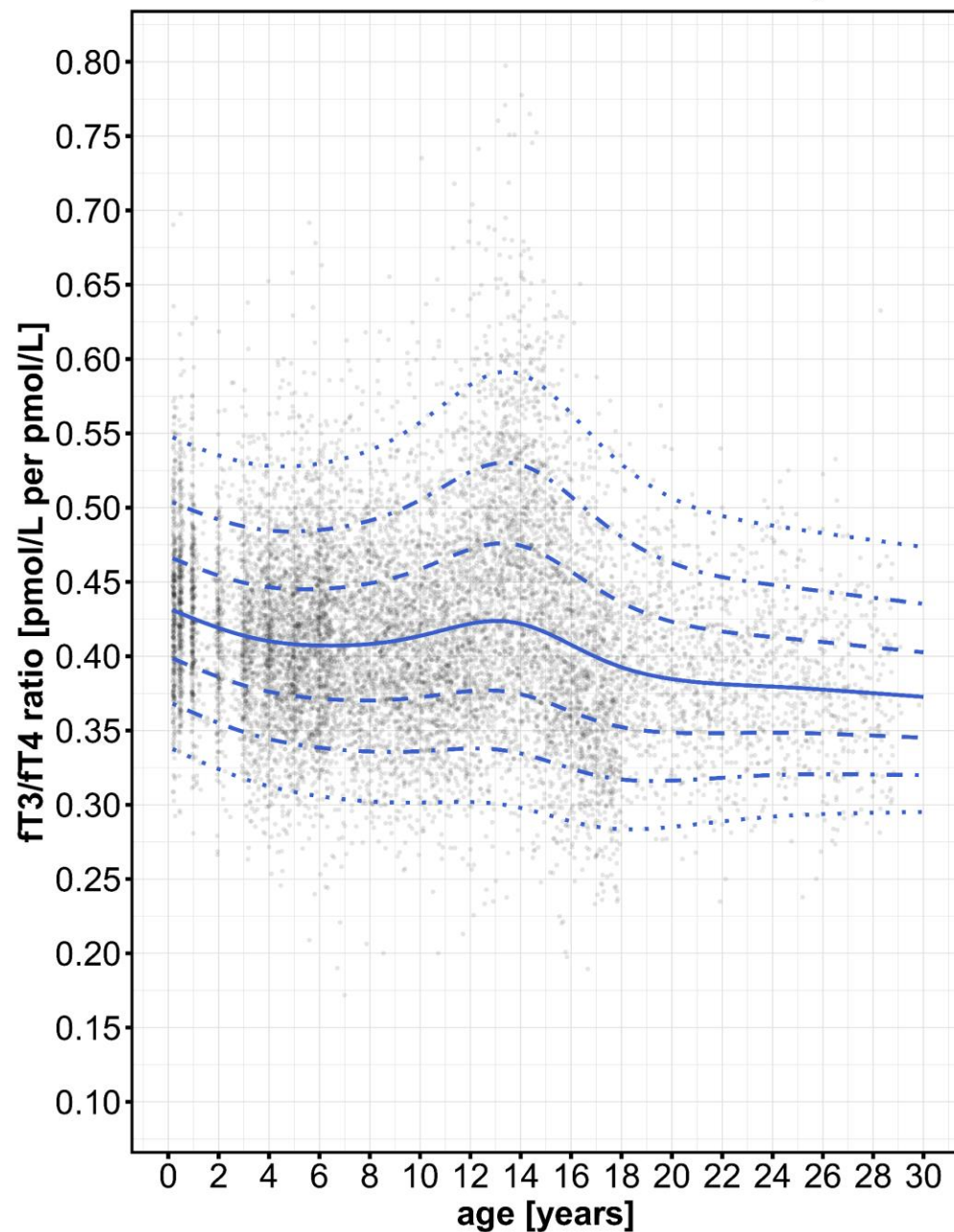

Supplement: Supplementary file 1 [file ijms-25-08585-s001.zip › Figure_S1.Distribution of the point clouds for the fT3fT4 ratios of all male and female control individuals.pdf]
